# Treatment of Inactive Ovaries of Holstein Dairy Cows by Epidural Injection of GnRH Analogue (Receptal) and Its Impact on the Reproductive Hormones, Oxidant/Antioxidant Profile and Micro and Macro-Elements Profile

**DOI:** 10.3390/ani13040653

**Published:** 2023-02-13

**Authors:** Yahia A. Amin, Alaa Eldin Z. Mahmoud, Rana A. Ali, Samer S. Fouad, Obeid Shanab, Rawia M. Ibrahim, Foad Farrag, Mustafa Shukry, Samah F. Ibrahim, Liana Fericean, Ragab Hassan Mohamed

**Affiliations:** 1 Department of Theriogenology, Faculty of Veterinary Medicine, Aswan University, Aswan 81528, Egypt; 2 Department of Theriogenology, Faculty of Veterinary Medicine, Sohag University, Sohag 82524, Egypt; 3 Department of Zoology, Faculty of Science, South Valley University, Qena 83523, Egypt; 4 Clinical Pathology of Veterinary Medicine, Qena University Hospital, South Valley University, Qena 83523, Egypt; 5 Department of Biochemistry, Faculty of Veterinary Medicine, South Valley University, Qena 83523, Egypt; 6 Division of Clinical Laboratory Diagnosis, Animal Medicine Department, Faculty of Veterinary Medicine, South Valley University, Qena 83523, Egypt; 7 Department of Anatomy and Embryology, Faculty of Veterinary Medicine, Kafrelsheikh University, Kafrelsheikh 33516, Egypt; 8 Physiology Department, Faculty of Veterinary Medicine, Kafrelsheikh University, Kafrelsheikh 33516, Egypt; 9 Department of Clinical Sciences, College of Medicine, Princess Nourah bint Abdulrahman University, P.O. Box 84428, Riyadh 11671, Saudi Arabia; 10 Department of Biology and Plant Protection, Faculty of Agriculture, University of Life Sciences “King Michael I”, 300645 Timișoara, Romania

**Keywords:** inactive ovaries, GnRH analogue, epidural injection, reproductive hormones, oxidant/antioxidant profile, and micro- and macro-elements

## Abstract

**Simple Summary:**

Ovarian inactivity is one of the most common ovarian diseases that cause infertility. Several trials were carried out to find a reliable treatment for inactive ovaries. In veterinary medicine, numerous pharmacological substances are delivered into the epidural area, such as local anaesthetics and analgesics. Using this approach, hormones and analogues can also be administered to produce a targeted local pharmacological response. Since sympathetic neurons from the ovarian plexus nerve and the hypogastric nerve innervate the bovine ovaries, the epidural injection may have an indirect impact on the ovaries. In the present study, the epidural injection of Receptal has the potential to induce estrus response and conception incidences in cows have ovarian inactivity. Progesterone, follicle-stimulating hormone, and luteinizing hormone concentrations were significantly increased in the cows treated with the epidural injection of Receptal, whereas testosterone and cortisol decreased following treatment. In addition, the treated cows had greater total antioxidant capacity and glutathione peroxidase concentrations than the non-treated cows. Serum concentrations of magnesium increased following receptal treatment, but differences in other minerals were not detected. This research suggests a novel, effective method of treating inactive ovaries with an epidural infusion of a gonadotropin-releasing hormone agonist.

**Abstract:**

This study was designed to evaluate a new therapeutic approach for inactive ovaries based on the epidural administration of a GnRH agonist (Receptal) and an investigation of the impact of this treatment on the hormonal, oxidant/antioxidant and micro- and macro-element profiles. Sixty cows with postpartum anestrus were divided into two groups: the first group (group R_epid_, n = 30) was administered an epidural injection of Receptal, while the second group (group C_epid_, n = 30) received saline and was considered the control group. Evaluation of hormonal (progesterone, FSH, LH, testosterone, and cortisol), oxidant/antioxidant (MDA, SOD, GPx and TAC) as well as micro- and macroelement (calcium, phosphorus, manganese and magnesium) profiles was done in serum. The results showed that the epidural injection of Receptal has the potential to induce estrus response and conception incidence in treated cows. Compared to the control group, progesterone, FSH, and LH concentrations were significantly increased in the treated group, whereas testosterone and cortisol decreased (*p* < 0.05) following treatment. In addition, the treated group had greater TAC and GPx concentrations than the control group. Serum concentrations of magnesium increased (*p* < 0.05) following receptal treatment, but differences in other minerals were not detected. This research suggests a novel, effective method of treating inactive ovaries with epidural infusion of a GnRH agonist.

## 1. Introduction

Ovarian diseases are one of the major causes of the high economic loss in dairy cows due to their major role in the induction of ovarian dysfunction and lower reproductive performance associated with their high incidence rate [1]. One of the most common ovarian diseases is postpartum ovarian inactivity, which is recorded to represent about 50% of the ovarian diseases [2] and is regarded as one of the main reasons for dairy cow infertility [2,3,4]. It is well known that transitory ovarian malfunction and lack of periodic follicular activity are the main causes of ovarian inactivity or inactive ovaries (IO) [5]. Therefore, the term “inactive ovaries” refers to ovaries that only produce follicles up to the point of follicular wave emergence, or up to 8 mm in diameter, when growth stops [6]. Previously, it was recorded that at the beginning of breeding programmes [7] or 63 days after calving [8], 20% of dairy cows were in anestrus and had inactive ovaries. As opposed to this, some other cows exhibit ovarian rebound and estrous cycles at the expected time following calving, and some may even have been inseminated, only to eventually regress back to having an inactive ovary [9]. Therefore, the condition by turn may lengthen calving to conception intervals and calving intervals [10].

Several studies were designed to find a reliable treatment for inactive ovaries. Some depend on natural components such as poly-herbal formulations [11], while others apply synchronization protocols using CIDR and GnRH injection [12], and others are concerned with the addition of nanoparticles or polymers to GnRH [13]. Mohammadzadeh et al. [14] reported that GnRH is a neuropeptide known to regulate reproduction in vertebrates. External injection of GnRH analogues causes a rapid release of LH after activating pituitary GnRH receptors, which finally causes luteinization [15]. Many mammalian extrapituitary tissues have been shown to express GnRH receptors (reviewed by Ramakrishnappa et al. [16]. In particular, Ramakrishnappa et al. [17] reported finding GnRH-receptor mRNA in the follicle and corpus luteum tissues of the bovine ovary. Additionally, GnRH receptors are distributed throughout the mammalian spinal cord at all levels [18]. These premises demonstrate the possibility that GnRH analogues may exert their effects through activation of extra-pituitary receptors in addition to pituitary ones [19]. In veterinary medicine, numerous pharmacological substances are delivered into the epidural area, such as local anaesthetics and analgesics [20]. Using this approach, hormones and analogues can also be administered to produce a targeted local pharmacological response. Since sympathetic neurons from the ovarian plexus nerve and the hypogastric nerve innervate the bovine ovaries [21,22], the epidural injection may have an indirect impact on the ovaries. Therefore, in this study, we investigate treatment of inactive ovaries by a new therapeutic treatment based on epidural injection of GnRH analogue called Receptal.

IO in cows can be developed and exacerbated by many factors, such as poor body condition score (BCS), insufficient feed intake, excessive milk production, and improper stress and disease management [23]. Similar to how it affects many other cells, oxidative stress, which results from an imbalance between the generation and elimination of ROS, directly harms the intra-ovarian environment. Additionally, all primary oocytes are developed by the 5th month of fetal life and are quiescent until meiosis I is finished, a lengthy process that makes the oocyte vulnerable to persistent oxidative stress [24]. To date, numerous studies have demonstrated that the piling up of ROS in the ovaries impairs egg quality, triggers granulosa cell (GC) apoptosis, and hastens corpus luteum degeneration [25,26,27,28]. Additionally, it impairs oocyte and GC communication, impairing preovulatory oocyte development [29]. Actually, lipid peroxidation cascades are responsible for oxidative damage to the ovary and negatively affect ovulation, meiosis, and folliculogenesis over time [30,31]. The exact processes that link ROS to female fertility are still mysterious. One study that looked at the connections between ROS, female fertility, and reproductive outcomes hypothesized that these links would be obscured by fluctuations in the estrous cycle that are considered typical [32].

During the first few weeks of lactation, high-yielding dairy cows frequently experience a period of negative energy balance (NEB) [33]. Changes in the blood metabolite and hormone profile, which characterize NEB status during early lactation, are critical for future health and fertility [34,35]. Moreover, it was discovered that a delay in postpartum ovarian activity in ruminants is thought to be caused by low blood levels of minerals, which also play an important role in the actions of hormones and enzymes [36]. Mineral element deficiencies, such as those in phosphorus (P), copper (Cu), and zinc (Zn), are linked to abnormally low fertility and anestrous conditions [37]. Changes in blood biomarkers like minerals have been utilized as diagnostic markers for various disorders for a few decades. For instance, blood calcium levels during the week before and up to immediately following calving have been used to predict postpartum illnesses [38,39]. To our knowledge, in dairy cattle, data about the roles of minerals in the development of postpartum ovarian activity suffers from a shortage.

The present study was designed to investigate the effects of applying a novel technique of treatment by epidural injection of GnRH analogue (Receptal) in inducing resumption of ovarian activity, inducing estrus, and pregnancy achievement in cows suffering from postpartum ovarian inactivity, as well as to determine the effects of this treatment on oxidative stress and the mineral profile of these diseased cows.

## 2. Material and Methods

### 2.1. Animals

All procedures were carried out in accordance with the guide approved by the Ethics Committee of the Faculty of Veterinary Medicine, South Valley University, Egypt (No 69/21 September 2022). The study was conducted on a dairy farm in Qena, Egypt, on 60 pluriparous Friesian cows suffering from inactive ovaries (3–5 years old, mean weight 350 kg and body condition score (BCS) ≥ 3) in post-partum from 90 to 120 days. Detection of BCS depends on a five-point scale of one (thin) to five (obese) [40]. Each cow was fed a total mixed ration (TMR) while being tethered in tie stalls. The TMR diet’s composition and chemical components were 1.03 kg cottonseed, 1.50 kg soybean husk, 2.50 kg alfalfa, 1.30 kg soybean meal, 2 kg corn flakes, 1 kg molasses, 25.37 kg silage, and 3.00 kg corn. The TMR used in this study was prepared according to NRC [41]. In addition, animals have unlimited access to water. Bovine diarrhea, brucellosis, bovine leucosis, and tuberculosis were all ruled out from the study, as were common parasites (coccidians and strongyloides). Cows with no signs of estrus for more than 90–120 days after calving were used in this study. When neither corpora lutea nor follicles were found on either ovary of a cow at two ultrasonography tests performed simultaneously with rectal palpation at 10-day intervals, the animal was deemed to have ovarian inactivity [42,43], in addition to progesterone measurements of concentrations <1 ng/mL).

Clinical reports contain full information about the clinical history of each cow in the study was prepared. Soon after parturition and for eight successive weeks, gynaecological examinations were conducted twice to record the reproductive state and/or abnormality. Ultrasonography was used to facilitate examinations.

The initial estrus and breeding of the cow after uterine involution occurred at 50 to 55 days postpartum, and the milk yield was noted at that time (average milk yield per day of 20 kg). Rectal examination and B-ultrasonic detection (SonoScape A5v Veterinary Ultrasound scanner; SonoScape Medical Corporation, Shenzhen, China) with a multifrequency linear transducer (5–12 MHz) were used to check for persistent corpus luteum, ovarian atrophy, corpus luteum cysts, follicular cysts, and uterine-related diseases. At initially, a teaser bull was used to detect the absence of estrous symptoms and make a tentative anestrous diagnosis. A case history and farm records were used in the selection of cows used in the study, depending on poor heat manifestations linked to long calving intervals.

### 2.2. Blood Samples and Experimental Protocol

Blood samples were taken by jugular venipuncture from all cows 10 days prior to the start of the treatment regimen. In addition, blood collection continued to occur on days 0, 7, and 15 of the regimen and on the day of estrus. Centrifugation of the samples occurred at 3000 rpm for 15 min. Preservation of the resultant serum occurred at –20 °C The chosen cows were randomly split into two groups: group I, with a total of 30 animals, underwent the treatment regimen, while group II, with a total of 30 animals, served as the control group and did not receive any therapy (saline) as the following:

- Group R_epid_ (treated group): 30 cows were administered GnRH agonist by epidural route in the sacrococcygeal space (5 mL Receptal per head, each 1 mL contained 0.004 mg Buserelin, Intervet, Holland).
- Group C_epid_ (control group): 30 cows were administered 5 mL of normal saline solution by epidural route in the sacrococcygeal space (0.9% NaCl).

Cows were observed for the expression of estrus daily after treatment was done. The clinical examination associated with transrectal ultrasound was performed every 2 days after treatment for 20 days to identify the initial onset of estrus. Estrus was identified by looking for the characteristic estrus symptoms (vulval oedema, clear mucous vaginal discharge, male receptivity) and by transrectal palpation, which revealed the presence of a preovulatory follicle. Cows observed in estrus were bred by natural service. Bred cows were observed for return to estrus. Forty-five days after serving, transrectal palpation and ultrasound were used for pregnancy identification. As a result, the first- and second-serving pregnancy rates were noted. A Fisher’s Exact Test was used in statistical analysis to examine the findings related to the induction of estrus and pregnancy rate. 

### 2.3. Determination of Hormonal Profile in Blood

Using competitive double-antibody enzyme immunoassays, progesterone (P4) and testosterone (T) were determined as previously mentioned [44]. Assay sensitivities were 0.391 pg/sample volume for P4 and 0.195 pg/sample volume for T. In addition, ELISA (enzyme-linked immunosorbent assay) kits were used to measure follicle-stimulating hormone (FSH) [45]. Luteinizing hormone (LH) determination was carried out using ELISA kits [46]. Using the radioimmunoassay method, the cortisol was assessed [47].

### 2.4. Determinations of Oxidant/Antioxidant Levels and the Mineral Profile in Blood

Using commercial ELISA kits (Cayman, USA), the levels of malondialdehyde (MDA), superoxide dismutase (SOD), and total antioxidant capacity (TAC) were assessed in accordance with the manufacturer’s recommendations. Glutathione peroxidase (GPx) was determined through diagnostic kits (Sigma Aldrich, St. Louis, MO, USA), according to the manufacturer’s instructions. The mineral profiles of calcium (Ca), phosphorus (P), manganese (Mn) and magnesium (Mg) were determined. A standard curve method was used for each element to quantitatively detect the researched elements. Thermo Scientific’s iCE 3000 series atomic absorption spectrophotometer was used to measure the specified components quantitatively [48].

### 2.5. Statistical Evaluation

The results were statistically evaluated using SPSS^®^ (SPSS^®^19, Chicago, IL, USA). To assess significant differences, a general linear model ANOVA with repeated measurements was utilized. A Fisher’s Exact test was used in statistical analysis to examine the findings related to the induction of estrus and pregnancy rate.

## 3. Results

All animals responded quite easily to epidural treatment. There were no adverse effects reported, and neither the doses we used nor the follow-up sessions revealed any signs of pain or discomfort in the cows. The findings for reproductive parameters are shown in Table 1. A highly significant estrus induction rate was found by the investigation (*p* < 0.05) after the application of epidural injection of Receptal in the R_epid_ group (26 of 30, 86%), and the average time from treatment to the onset of estrus was 7 ± 1.27 days. With a conception rate of 88%, 23 cows successfully became pregnant; however, 12% of the cows were unable to conceive. None of the cows in the control groups (C_epid_) came into estrus during the study period (Table 1).

**Figure 1:**
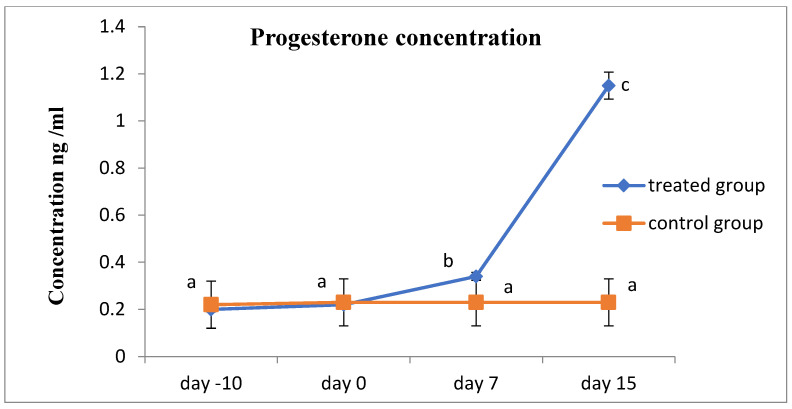
Serum progesterone concentration measurements (mean ± SE) in the epidural treated anestrous cows compared to those in the control group. Values with different letters (a–c) differ significantly (*p* < 0.05).

**Figure 2:**
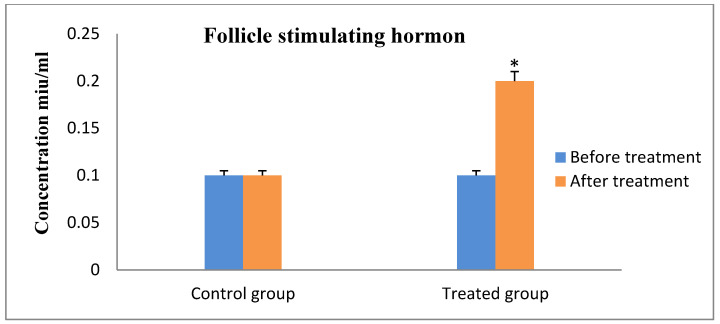
Serum follicle stimulating hormone concentration measurements (mean ± SE) in the epidural treated anestrous cows compared to those in the control group. Values with asterisk (*) differ significantly (*p* < 0.05).

**Figure 3:**
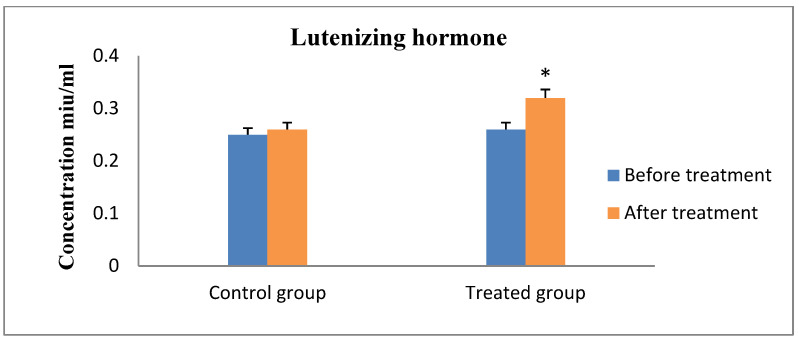
Serum lutenizing hormone concentration measurements (mean ± SE) in the epidural treated anestrous cows compared to those in the control group. Values with asterisk (*) differ significantly (*p* < 0.05).

**Figure 4:**
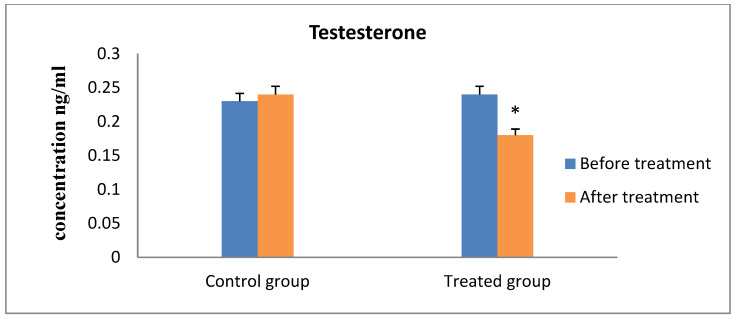
Serum testosterone hormone concentration measurements (mean ± SE) in the epidural treated anestrous cows compared to those in the control group. Values with asterisk (*) differ significantly (*p* < 0.05).

**Figure 5:**
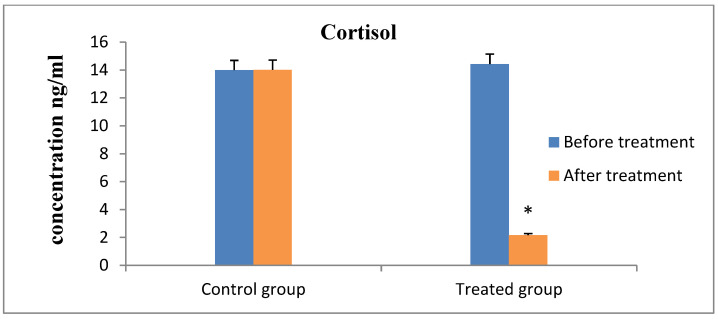
Serum cortisol hormone concentration measurements (mean ± SE) in the epidural treated anestrous cows compared to those in the control group. Values with asterisk (*) differ significantly (*p* < 0.05).

Progesterone values indicated that all of the cows were experiencing ovarian inactivity, with serum progesterone levels being less than 1 ng/mL prior to the study, according to the results of hormonal analysis. As the animal responded to therapy and exhibited estrus, which was followed by ovulation and pregnancy, the concentration increased considerably (Figure 1). On the contrary, the serum FSH and LH concentrations showed significantly increased values after receiving therapy and the animals exhibit estrus in the R_epid_ group compared to before treatment and the control group (Figure 2 and Figure 3). The serum testosterone and cortisol levels were significantly decreased after treatment in the R_epid_ group compared to before treatment and the control group (Figure 4 and Figure 5). The inter- and intra-assay coefficients of variation were 14.9% and 3.9% for P4, and 7.1% and 7.4% for T, respectively.

Table 2 shows the serum concentrations of MDA and SOD in both groups. The serum MDA and SOD concentrations in the R_epid_ group did not differ after Receptal treatment compared to the C_epid_ group and before treatment. GPx and TAC concentrations (mmol/L) exhibit increasing trends after epidural treatment in the R_epid_ group. These trends were significant (*p* < 0.05) when the cows came into estrus, whereas the TAC concentrations were almost static in the cows of the control group.

The mean serum Ca, P and Mn values in the R_epid_ group did not differ after receptal epidural treatment compared to the C_epid_ group and before treatment. In contrast, magnesium concentrations (mmol/L) exhibit a significant difference after epidural treatment in the R_epid_ group. The difference is represented by a significant (*p* < 0.05) increase when the cows came into estrus, whereas its concentrations were almost static in the cows before treatment and in those of the control group (Table 3). 

## 4. Discussion

To the best of our knowledge, this is the first comprehensive research examining the use of the GnRH analogue Receptal, administered epidurally, to treat inactive ovaries in dairy cows. The current experiment shows that epidural administration of Receptal is an efficacious treatment for ovarian inactivity in bovine species. Epidural injection induces ovarian resumption and induces estrus in a higher percentage of cows, demonstrating its effectiveness when given in this way. The efficacy of epidural injections of hormones for the treatment of reproductive disorders has been reported in several studies. According to Sakaguchi et al. [49], a single epidural injection of FSH was sufficient to cause super-ovulation and result in a greater FSH level than several injections in Holstein dairy cows. Lecirelin, a GnRH analogue, was shown to be efficacious in treating follicular cysts in dairy cows by Annalisa et al. [19]. In addition, induction of ovulation in female dromedary camels was also achieved by epidural administration of the GnRH agonist (Lecirelin) [50]. The findings of our study are consistent with those of the previous studies that also confirmed the efficacy of the epidural route of hormonal injection in the treatment of reproductive problems without inducing any side effects.

According to Annalisa et al. [19], the outcome is likely the result of a synergistic interaction between GnRH agonist systemic absorption and its effects on sites other than the hypophysis, such as the spinal cord and/or ovary. In reality, GnRH agonists may have affected the pituitary gland via the epidural canal, the spinal cord, and/or the ovary, as well as through systemic diffusion and subsequent effects on the hypophysis. GnRH receptors were seen in the ovine spinal cord by Dolan et al. [18]. We can therefore hypothesize that Receptal may have caused a Ca2+ influx in the neurons by acting on the spinal GnRH receptors [15], causing norepinephrine release from the sympathetic terminals of neurons innervating the ovary [21]. Norepinephrine plays a role in the development of luteal tissue after enhancing follicular wall contractility, an event useful for rearrangement of post-ovulatory granulation tissue [51,52]. This indicates that follicular maturation, steroid secretion, and ovulation are influenced by the sympathetic innervation [53,54].

Additionally, when given epidurally, the Receptal might go through the cerebrospinal fluid to the central nervous system, where it would then travel to the hypothalamus and hypophysis [18,55,56]. We can therefore hypothesize that Receptal, when given epidurally, is thought to activate GnRH receptors in places other than the hypohysis in order to obtain a better reaction (functional synergism of action) [19].

In the current study, investigation of the hormonal profile of inactive ovaries of cows revealed that reproductive hormones including progesterone, FSH and LH exhibit lower levels. This corresponds to the recognized hormonal traits of inactive ovaries [57]. Receptal, a GnRH analogue, was administered epidurally to treat inactive ovaries in cattle, and this greatly increased the secretion of P4, FSH, and LH. The epidural dose of Receptal resulted in a high concentration of P4 of > 1 ng/mL, which demonstrated luteal activity and was compatible with the other findings of the estrus response and ultrasonographic exams in this investigation. Therefore, ovulation is confirmed by the high P4 level, which is produced by the corpus luteum resulting from an ovulated follicle. These outcomes are consistent with recent research that illustrated that animals with inactive ovaries demonstrated a significant decrease in serum progesterone values [11,12,58], which agrees with the results of previous researchers [59,60].

In the present study, FSH levels were low in cows with ovarian inactivity before treatment. Later, after treatment by epidural injection, the FSH levels increased. These findings are in line with Sakaguchi et al. [49] who compare the effect of a single epidural injection of FSH in dairy cows with its twice intramuscular injection in a trial to induce superovulation. According to the authors, plasma FSH concentrations were greater and were stable for at least 4 days following an epidural FSH injection than they were following the standard technique of several intramuscular injections. However, other authors stated some alterations in these findings. According to a previous study, the plasma concentration of FSH in the epidural group (single dose of epidural injection) increased dramatically within 2 h compared to the control group, which received FSH twice daily by intramuscular injection, before significantly declining thereafter (*p* < 0.01) and not remaining above baseline after 10 h of administration. Unlike the epidural group, the control group’s FSH level was consistently kept in the circulation for 72 h [61].

Pituitary FSH is necessary for ovarian follicle growth and maintenance in both single-ovulating and multiple-ovulating species [62]. FSH in the blood was found to be significantly lower in the cows with smooth inactive ovaries than in normal cyclic cows [63]. Normal cyclicity is initiated by GnRH release, which also induces the anterior pituitary to release FSH and LH. LH and FSH exert a direct influence on the ovary to promote follicular development and maturation, which results in estrus. Therefore, gonadotropins (FSH and LH) represent the main factors that ovarian follicle development is dependent on for growth and function. In addition, the switch to gonadotropin reliance in cattle occurs in 1–3 mm antral follicles, and subsequent follicle development and growth depend on sufficient circulating FSH levels [64,65]. FSH interacts with numerous signalling pathways and performs a variety of tasks within the follicle that support the development and quality of the oocyte as well as the function of the ovulatory follicle. The ability of ovulatory-size follicles to respond to LH determines how they develop later and how fully they mature in preparation for ovulation [66,67]. The ovulatory follicle undergoes a transformation after ovulation known as luteinization, which is controlled by LH and results in the formation of the corpus luteum and a concurrent switch from estradiol to progesterone production [68].

In the current investigation, the epidural therapy resulted in a somewhat lower serum testosterone levels than the control treatment. In bovine granulosa cells, FSH facilitates the conversion of testosterone into E2 [69], and in the current investigation, the blood content of FSH was higher with the epidural therapy than with the control treatment. We predict that E2 concentrations in developing follicles may be higher with the epidural therapy than with the control treatment, despite the fact that serum E2 concentrations may not have significantly varied between groups at the systemic level. This prediction depends on the findings reported by other investigations that showed similar peripheral E2 concentrations were recorded in association with varied intrafollicular E2 concentrations [49].

Chronic and acute stress can trigger disruptions in reproductive function. Acute stress negatively impacts ovarian cyclicity by interfering with the normal hormone release from the hypothalamic-pituitary-gonadal axis, which regulates reproduction [70,71]. In the present study, the serum concentration of cortisol was higher in cows with ovarian inactivity. The high cortisol level indicates that these cows might suffer from an unknown type of stress that could be related to the disease itself or to the temperature of the weather. As the cortisol concentration was lower with the epidural treatment than with the control cows, this reveals that the stress might result from the disease itself. However, it is well known that heat stress can increase cortisol [72]. Therefore, it was preferable to record the study site’s daily temperature; nevertheless, only the Qena Meteorological Report was created, which can be viewed as a study constraint. 

Reactive oxygen and nitrogen species (ROS and RNS) are produced as a result of heat stress, pregnancy, and milk production. These ROS and RNS, which also affect lipid peroxidation, apoptosis, and fertility, include hydroxyl radicals, superoxide ions, hydrogen peroxide, and nitric oxide radicals [73]. This indicates that free radical chain reactions are the main component of the biological consequences of the ROS and RNS and are responsible for inducing infertility by affecting folliculogenesis, steroidogenesis, and the pre-implantation of an embryo, which are sensitive to free radical damage [74]. Evidence is mounting that oxidative stress is one of the primary triggers of mammalian oocyte and granulosa cell (GC) apoptosis [25,75]. Evaluation of the oxidant and antioxidant profiles has been described well in buffaloes suffering from ovarian inactivity, but its evaluation in dairy cows suffering from the same disease remains limited. Therefore, we will compare the findings of the current study with the available data about cows and buffaloes that have ovarian inactivity.

In the present study, investigation of the oxidative profile revealed that the levels of the oxidative stress biomarker MDA in cows with ovarian inactivity before and after treatment did not exhibit a significant difference. A previous study reported that no significant difference was found in the levels of MDA before and after treatment with a polyherbal formulation in cows with inactive ovaries [11]. Contrary to this, another study in buffalo stated that MDA concentrations in the blood of acyclic buffaloes were higher before treatment by CIDR and that later the concentration significantly decreased when the buffaloes came into estrus [12]. It is important to mention that the latter study was carried out on summer-stressed anestrous buffalo. This indicates that heat stress might play a role besides ovarian inactivity in leading to an increase in its value. 

The antioxidant profile check in the present study reveals that the levels of SOD are higher after treatment compared to before treatment and in control cows. Through spontaneous dismutation, SOD transforms ROS produced by the cell into hydrogen peroxide [76]. These findings concur with those documented in a previous study [77], in which plasma SOD levels in normal cyclic and postpartum anestrous (PPA) animals were not significantly different from one another. However, the latter authors discovered that erythrocytic SOD was considerably higher (*p* < 0.01) in the regular cyclic animals than it was in the PPA animals. This shows that ovarian inactive cows are under oxidative stress, and the increase in erythrocytic SOD values was likely brought on by enhanced erythrocytic turnover for the protection of oxidative damage. While plasma SOD did not differ significantly between the two groups as it indicates a short-term stress status [77].

The current study revealed that GPX-3 and TAC levels were significantly different in both groups and before and after treatment in the epidural treated group. These findings are consistent with the earlier investigation [78], in which the authors reported that the group of cows with inactive ovaries was found to have a difference that is statistically significant (*p* < 0.05) in TAC compared to the normal cyclic group. Likewise, a similar report for dairy buffaloes was also documented in related experiments [79], in which the concentration of the TAC indicator in serum was significantly lower (*p* < 0.001) in buffaloes suffering from ovarian inactivity when compared to regularly cycling buffaloes. In addition, another study mentioned that TAC was significantly increased in cows with inactive ovaries following treatment with CIDR and prostaglandin F2 α (PGF2α) compared to the non-treated group [12]. Furthermore, a recent study in dairy cows stated results almost similar to those found in the present study, in which TAC levels were found to be increased after polyherbal treatment of ovarian inactivity, but their increase was not significant [11]. According to a recent paper by Jan et al. [80], it is now clear that oxidative stress is responsible for the estrous cycle’s disruption, as the results of the experiment showed that the follicular fluid of anestrous buffaloes had generally greater amounts of oxidative stress indicators together with lower levels of total antioxidant capacity (TAC).

In the current study, the lower levels of antioxidants (GPx, and TAC) in cows suffering from inactive ovaries indicated that these cows were under oxidative stress. In addition, as the function of the antioxidants is to both stop the production of free radicals and neutralize their effects, the drop in the levels of these enzymatic antioxidant values was likely caused by an increase in turnover for the protection of oxidative damage [81]. It is well known that reducing stress production is necessary to sustain female animals’ reproductive effectiveness [82]. Therefore, in order to maximize cows’ performance, it is advised that, in addition to regular feeding, extra supplementation of antioxidant vitamins at times of stress can be helpful [77].

In terms of nutrition, dairy cattle’s reproductive health depends on maintaining appropriate levels of both macro- and microelements [83,84]. Our current results show that cows with inactive ovaries have non-significant (*p* > 0.05) variations in calcium and phosphorus levels before and after treatment. A recent study by Wang et al. [85] pointed out the same results, in which Ca and P exhibited non-significant changes between cows in estrus compared to cows with inactive ovaries. Furthermore, a previous study also mentioned the same findings, which illustrated that non-significant differences in the blood concentrations of calcium and phosphorus between the cyclic and non-cyclic cows were found [86]. Calcium appears to have an indirect impact on animal reproduction. It is one of the key minerals that are known to affect how well an animal can utilize other trace elements. Its impact on specific enzyme systems may be mediated via interference with reproductive effectiveness [87]. Others agreed, stating that calcium is essential for increasing ovarian preovulatory follicle size and number as well as ovulation rate [88,89]. According to Virmani et al. [87], acyclic animals’ low calcium levels are caused by either a failure to maintain a normal calcium level due to metabolic abnormalities or by increased calcium excretion. Phosphorus is necessary for the transfer of biological energy, especially through adenosine triphosphate. A lack of phosphorus can prevent fertilization from occurring, which can lead to early embryonic mortality and repeat breeding as well as anestrus conditions in animals [90].

Devkota et al. [91] reported that no buffaloes became pregnant within 4 months after treatment with PGF2α, GnRH, or vitamin-mineral (Vit-M) supplements in anestrus buffaloes when the levels of phosphorus were lower than 4.5. In addition, the authors indicate that the pregnancy rate reached a high level in cows that had high phosphorus (more than 4.5). These outcomes are in line with what we have seen. Interestingly, cows suffering from ovarian inactivity in the present study had phosphorus concentrations higher than 4.5 mg/dL (9.51 mg/dL). The results of the present investigation, however, do not coincide with those of Song et al. [2], who found that the calcium and phosphorus concentrations in the plasma of cows with inactive ovaries were significantly lower than those in healthy control cows. The hypothesis that low calcium and phosphorus levels contribute to the development of inactive ovaries was mentioned by the latter authors, who also added that fat mobilization caused by NEB in early lactation is also responsible, along with Ca and P, for the development of static cases of the ovary. Nonetheless, the same authors encourage the need for more investigation into the synergy mechanisms between these two factors related to the development of inactive ovaries.

Although nutritional status is frequently acknowledged as a substantial contributor to inactive ovaries because postpartum ovarian activity and energy balance are closely related [92,93], the results of the current study reveal that calcium and phosphorus levels are almost in the normal range and are not responsible for the incidence of ovarian inactivity. This suggests that hormonal disturbance and oxidative stress may have a major role in the occurrence of ovarian inactivity in the current study. Therefore, further research needs to be performed to determine the mechanisms behind inactive ovaries.

Manganese and magnesium have yet to be described in relation to the inactive ovaries of cows; therefore, we investigated their levels in cows with inactive ovaries and compared them with their levels after treatment when the cows become healthy and come into estrus. Few studies have particularly examined the impact of manganese on reproduction and metabolism in dairy cows, despite the fact that Mn is a vital component of a number of enzymes involved in antioxidant protection, including superoxide dismutase, as well as carbohydrate and lipid metabolism [94]. Additionally, decreased reproductive success was accompanied by a loss of appetite brought on by a magnesium deficit [95]. In the current study, a non-significant (*p* > 0.05) decrease in manganese level was observed in cows that have ovarian inactivity compared to its level when the cows come into estrous after treatment by epidural injection of Receptal. This result is in line with the result reported in a previous study that serum manganese levels exhibit no differences between ewes and does with static ovaries compared to cyclic ones [96]. Manganese functions as a co-factor in the synthesis of cholesterol, which is required for the production of steroid hormones such as testosterone, progesterone, and estrogen [97]. High E2 levels have positive feedback with the hypothalamus, which triggers the release of GnRH and, ultimately, ovulation. Therefore, deficiency causes poor fertility problems in both male and female. According to Wilson et al. [98], manganese insufficiency was influenced by conditioning factors, particularly the ration’s calcium and phosphorus levels. The primary condition caused by manganese deficiency is infertility, which is accompanied by quiet estrus, delayed ovulation, an irregular estrous cycle, cystic ovarian tissue, and poor follicular development [99,100].

In the current study, a significant increase in the magnesium level was observed in the cows that came into estrus after treatment by epidural injection of receptal. Although magnesium typically has no direct effect on the reproductive status of animals, its disturbance causes a big problem in the reproduction process. This importance of magnesium results from the fact that magnesium and calcium continue to have an almost antagonistic relationship in the body and any disturbance in the Ca-P-Mg balance can have an impact on reproduction. Moreover, magnesium deficiency leads to loss of appetite, which subsequently causes reduced reproductive efficiency [95]. This outcome does not align with the findings of earlier investigations that serum magnesium levels exhibit no differences between cows with inactive ovaries compared to cyclic ones [85].

## 5. Conclusions

Epidural administration of Receptal enables it to enter the circulation; however, it is also plausible that a more direct action on the spinal cord and ovaries happened, causing the resumption of ovarian function by local processes other than LH surge triggering. Given our findings, this study establishes the foundation for a novel therapeutic strategy for the treatment of inactive ovaries based on the epidural administration of a GnRH agonist, with the goal of facilitating the restoration of ovarian activity, particularly in situations where traditional therapeutic strategies have failed. Additionally, analysis of the mineral profile and oxidant/antioxidant characteristics of anestrous cows may aid in understanding this reproductive issue. To determine the potential benefits of employing the epidural administration route over traditional ways, more research is required.

## Figures and Tables

**Table 1:** Reproductive parameters (estrus, pregnant cows after 1st and 2nd serving, and total pregnancies) in Group R_epid_ (cows administered Receptal by epidural route) and Group C_epid_ (cows administered saline solution by epidural route).

Groups	No. of Cows	Estrus	Pregnant Cows after 1st Serving	Pregnant Cows after 2nd Serving	Total Pregnancies
Treated group	30	26 (86%)	18 (69%)	5 (63%)	23 (88%)
Control group	30	0 (0)	0 (0)	0 (0)	0 (0)

**Table 2:** Comparison of oxidative/antioxidant biomarkers in group R_epid_ (cows administered Receptal by epidural route, treated group), and group C_epid_ (cows administered saline solution by epidural route, control group).

Parameters	Groups	Before Treatment	After Treatment
Malondialdehyde (nmol)	Treated group	4.8 ± 0.12	5.2 ± 0.11
	Control group	4.7 ± 0.11	5.1 ± 0.13
Super Oxide Dismutase (U/mL)	Treated group	386.5 ± 0.1	401.7 ± 0.12
	Control group	387.5 ± 0.13	400.1 ± 0.14
Glutathione peroxidase (U/L)	Treated group	1335 ± 0.11	1382 ± 0.15 *
	Control group	1336 ± 0.12	1337 ± 0.14
Total Antioxidant Capacity (mM)	Treated group	3.6 ± 0.23	4.6 ± 0.25 *
	Control group	3.5 ± 0.21	3.7 ± 0.26

Values are expressed as mean ± SE. Asterisks (*) indicate statistically significant (*p* < 0.05).

**Table 3:** Comparison of the mineral profile of group R_epid_ (cows administered Receptal by epidural route, treated group) and group C_epid_ (cows administered saline solution by epidural route, control group) (mean ± standard error).

Parameters	Groups	Before Treatment	After Treatment
Calcium (mg/dL)	Treated group	4.73 ± 0.07	3.64 ± 0.12
	Control group	4.51 ± 0.19	4.22 ± 0.17
Phosphorus (mg/dL)	Treated group	10.20 ± 0.12	9.51 ± 0.11
	Control group	10.21 ± 0.23	10.01 ± 0.25
Manganese (μg/L)	Treated group	3.22 ± 0.24	3.61 ± 0.23
	Control group	3.11 ± 0.16	3.22 ± 0.12
Magnesium (mg/dL)	Treated group	1.43 ± 0.09	2.23 ± 0.08 *
	Control group	1.44 ± 0.01	1.45 ± 0.06

Values are expressed as mean ± SE. Asterisks (*) indicate statistically significant (*p* < 0.05).

## Data Availability

Not applicable.
